# RPC-Lex: A dictionary to measure German right-wing populist conspiracy discourse online

**DOI:** 10.1177/13548565221109440

**Published:** 2022-06-21

**Authors:** Cornelius Puschmann, Hevin Karakurt, Carolin Amlinger, Nicola Gess, Oliver Nachtwey

**Affiliations:** 9168 University of Bremen, Germany; 27209 University of Basel, Switzerland; 27209 University of Basel, Switzerland; 27209 University of Basel, Switzerland; 27209 University of Basel, Switzerland

**Keywords:** Automated content analysis, conspiracy theories, dictionary, Germany, political communication, right-wing populism, social media

## Abstract

We describe a novel computational dictionary for the study of right-wing populist conspiracy discourse (*RPC*) on the internet, specifically in the context of contemporary German politics. After first presenting our definition of conspiracy discourse and grounding it in antecedent research on mediated rhetoric at the intersection of right-wing populism and conspiracy theory, we proceed by outlining our approach to dictionary construction, relying on a combination of manual and automated methods. We validate our dictionary via parallel manual coding of 2,500 sentences using the categories contained in the dictionary as labels and compare the consensus result with the label assigned to each sentence by the dictionary, achieving satisfactory results. We then test our approach on two different datasets composed of alternative news articles and Facebook comments that spread conspiracy theories. Finally, we summarize our observations both on the methodological premises of the approach and on the object of populist right-wing conspiracy discourse and its dynamics more broadly. We close with an outlook on the potentials and limitations of the dictionary-based approach and future directions in applications of content analysis to the study of conspiracy discourse.

## Introduction

Conspiracy theories are frequently deployed by fringe political actors that aim to undermine political institutions and boost their own claims to legitimacy. Examples on the right include individual politicians, parties and movements (Engesser et al., 2017; Gerbaudo, 2018; Puschmann et al., 2020). Conspiracy theories relating to controversial political issues in particular appear to activate supporters of both right-wing and left-wing causes by tapping into strong emotions, such as alienation, fear and resentment (Hameleers, 2021). Indeed, conspiracy theories serve as a common denominator to ideologically disparate populist strands (Bergmann and Butter, 2020), while the intersection with right-wing populism is especially relevant to the German context (Dostal, 2015; Lees, 2018; Vorländer et al., 2018).

It therefore seems desirable to develop computational tools for the detection and measurement of the prevalence of such ideas in online environments (e.g. Gründl, 2020, for right-wing populism). Such tools are able to utilize the entanglement of conspiracy theories and language, that is, identify a conspiratorial vocabulary and the specific linguistic markers that signal an affinity for conspiratorial thinking. While the term *conspiracy theory* is extensively used in both societal discourse and academic literature, it has been widely pointed out that conspiracy theories, while often practicing a mimicry of science, do not generally take a form that would hold up to scientific standards and in most cases hardly constitute *theories*. Alternative terms such as ‘myth’ or ‘narrative’, that stress the affinities to religious belief systems or questions of form and narrative representation, have accordingly been proposed. We use the term *conspiracy discourse* in order to emphasize the communicative nature of conspiracy theories, the fact that, online in particular, conspiratorial ideas are told to be spread and developed, and that specific linguistic properties, from vocabulary to certain stylistic markers, reliably signal conspiracy communication.

Our paper describes RPC-Lex, a computational dictionary to measure the composition of right-wing populist conspiracy discourse (RPC) in German-language texts.^1^ By applying our dictionary to Facebook comments and alternative news media articles, we provide a cross-platform perspective within which we put our conceptual frame of RPC discourse to the test. As digital environments in general and social media in particular do not primarily cater to single conspiracy theories but are prone to the merging of conspiracy and other discourses, the investigation of common tropes of conspiracy thinking and right-wing populism offers distinct advantages, taking into consideration content as well as linguistic elements. Especially because of a recent flurry of research on both conspiracy theories and populism, a study of how different dimensions of these discourses intertwine is relevant for further theoretical and conceptual advancements. RPC-Lex as a resource is intended to further the study of new media technologies in a cross-platform perspective, applying an original conceptual and theoretical approach in order to study RPC-discursive elements and particular combinations in which they occur.

We first detail our theoretical approach and its implications for dictionary design by individually examining elements of conspiracy discourse and elements of right-wing populism and discussing the connection of both. These theoretical considerations then form the basis of the 13 categories of our dictionary, ranging from scandalization and anti-elitism to apocalypse and protest. After detailing our hybrid deductive-inductive design process for populating the categories, we present applications of the dictionary to two different use cases: a large body of user comments from a number of closed-membership Facebook groups and articles scraped from a set of popular German-language alternative news sources. We close by discussing the limitations of our approach and giving an outlook regarding future development of the resource.

## Right-wing populist conspiracy discourse and the German context

### Elements of conspiracy discourse

Research on conspiracy theories must contend with the difficulty of finding a clear-cut definition of the term. This proves challenging, first, because *conspiracy* is not a neutral term, but often used pejoratively to delegitimize others in public or private discussions. Second, because conspiracies do actually exist, and third, because, unlike the phenomenon itself, the term *conspiracy theory* is relatively new and has only been coined in the 1970s (Butter and Knight, 2020: 3–4). As Andrew McKenzie-McHarg noted, there is ‘a vagueness that inheres to conspiracy theory as a concept’, which opposes a simple and universally valid definition (2020: 16). Within this vagueness, still, there are elements common to conspiracy belief that are widely accepted in the field. The three basic characteristics of most conspiracy theories, as they have been coined by Michael Barkun, are that (1) ‘Nothing happens by accident’, (2) ‘Nothing is as it seems’ and (3) ‘Everything is connected’ (2013: 3–4). These premises are embedded in a broader mechanistic understanding of history as intentionally and secretly determined by a (small) powerful group of conspirators, able to control the course of events over long periods of time (Butter, 2020: 21). This worldview goes hand in hand with the underlying Manichean dualism between good and evil found in conspiracy theories (Cubitt, 1989: 15). Within this framework, a typology of conspiracy theories differentiates, first, ‘top-down’ from ‘bottom-up’ conspiracies, depending on where in the (national/social/political) hierarchy the imagined conspirators are located (e.g. the political elites as conspirators vs., for example, dissidents, socialists or Jews as conspirators). Second, ‘internal’ conspiracies are distinguished from ‘external’ ones, depending on whether the imagined conspirators are situated inside or outside of a nation’s institutions (e.g. leading politicians as internal conspirators vs. foreign intelligence services or terrorist organizations as external conspirators). While historically speaking bottom-up conspiracy theories have been very popular, in recent years, ‘there has been a growing tendency in the Western world to identify internal and top-down conspiracies’ (Butter, 2020: 14–16).

These general characteristics already provide some insight into shared content elements especially of top-down and internal conspiracy theories (e.g. in-group/out-group effects, criticism of elites, mistrust in public service news outlets). By using the term *conspiracy discourse* rather than conspiracy theory, we stress our interest in studying communicative practices and linguistic forms pertinent to conspiracy theories, following the premise that the collective experience of consuming and producing/updating (i.e. ‘prosuming’) is elementary to the evolution, spread and success of conspiracy theories in an online environment (Aupers, 2012: 27; Harambam and Aupers, 2014: 471; Madisson and Ventsel, 2021: 32–33; Stano, 2020: 484–486). Studying the contents of this communication is possible by identifying stylistic and content elements of such a conspiracy discourse and measuring key vocabulary corresponding with these elements (Maerz and Puschmann, 2020: 45–47). For these reasons, the term ‘discourse’ is better suited than ‘theory’ in order to study the prevalence, distribution and relationship of elements of conspiracy communication.^2^ By focusing on conspiracy discourse, ‘the references and correspondence to reality of particular versions of conspiracy theories are not particularly relevant. Rather, the focus is on the rhetorical and argumentative lines often repeated in conspiracy theories’ (Madisson and Ventsel, 2021: 41). While we do not present and study an exhaustive list of all elements characteristic for conspiracy discourse, we focus on certain core categories (presented throughout the paper in small caps) that (1) can be theoretically supported and (2) are especially relevant for our corpora, which largely stem from right-wing populist online environments (see Table 1 for an overview of all RPC-Lex categories introduced below).

**Table 1.** Overview of RPC-Lex categories with minimal definitions and numbers of entries.

	No.	Category	Number of dictionary entries
**Style**	1	Scandalization Vocabulary emphasizing conflict in order to engage readers throughoutrage and polemicize complex subjects	2449
	2	Suspicion/manipulation Vocabulary displaying and producing the constant suspicion ofbeing manipulated by politics and ‘mainstream media’	1266
	3	Exposure/revelation Vocabulary used to produce evidence by exposing supposed fakes,lies and propaganda as well as revealing the supposed truth of thealternative knowledge	1323
	4	Markers of distance Vocabulary and stylistic markers indicating contents and conceptsthat are rejected by the person using such vocabulary	288
**Antagonists**	5	Antisemitism Vocabulary of stereotypical perceptions of Jewish people that arefound in reciprocal interaction with conspiracy theories	625
	6	Anti-elitism Vocabulary indicating hostility or mistrust towards the political establishment and politicians, the ‘mainstream media’ and journalists, toward science and scientists, following a belief that these elites work against the interests of ‘the people’	1570
	7	Anti-immigration/Islamophobia Vocabulary indicating the xenophobic fears within conspiracist and right-wing populist discourses, construing immigrants and Muslims as central external threats to the white, Christian nation	1440
	8	Anti-gender/anti-feminism Vocabulary targeted at feminism and progressive ideas about gender aselitist agendas supposedly aimed at destroying the nuclear family,conservative values and masculinity/femininity in general	521
**Topoi**	9	Conspiracy General vocabulary of metaphors prevalent in conspiracy discourse,as well as vocabulary of specific, wide-spread conspiracy theoriesespecially relevant for RPC contexts	1176
	10	Apocalypse/downfall Vocabulary referring to the always impending yet never arrivingapocalypse, amplifying the initially vague threat to the nation into moregeneral fears of systemic downfall and extinction narratives	707
	11	Protest/rebellion Vocabulary calling for or justifying the (violent) defense of ‘the people’ against internal and external threats	922
	12	Nationalism (GER) Vocabulary aimed at the patriotic valorization of the (German) nation inneed of protecting, in stark opposition to both the construedthreat of immigrants and Muslims and the supposedly corrupt elites ofthe country	1169
	13	Esotericism Vocabulary of the strand of esotericism linked to the fascist ideology of adivine order of the peoples of the world, as well as more naiveesoteric terminology pertaining, for example, to conspiracist beliefsabout conventional medicine	649
		**TOTAL**	**14,105**

Elementary, the bolstering of an in- and out-group distinction prevalent in conspiracy discourse often takes the form of anti-elitism, as linguistic studies have shown (Keilholz and Obert, 2018; Schäfer, 2018). While this has not always been the case, ‘[b]laming those already in power […] became the dominant mode of conspiracy theorising in the West during the twentieth century’ (Butter and Knight, 2020: 3). Anti-elitism in its contemporary form includes a hostility toward science (Keilholz and Obert, 2018) and toward the ‘mainstream media’, which is believed to be controlled by corrupt elites who are trying to annihilate freedom of opinion, culminating in the historically loaded buzz word of the *Lügenpresse* (the ‘lying press’) (Gadinger, 2019; Koliska and Assmann, 2019; Lilienthal and Neverla, 2017; Seidler, 2016). Political elites, scientists and journalists are all part of the ‘establishment’ conspiracy believers aim to subvert (Engesser et al., 2017).

Closely linked to this anti-elitist dimension of conspiracy discourse are several linguistic categories. First, a vocabulary of suspicion/manipulation, displaying the constant suspicion of being manipulated by the ‘establishment’ (Keilholz and Obert, 2018: 211; Stumpf and Römer, 2018). Second, vocabulary of exposure signaling that something is exposed as fake, a lie, or propaganda and vocabulary of revelation signaling the production of evidence for the ‘alternative’ knowledge of the conspiracy theory, revealing an ‘actual truth’ (Keilholz and Obert, 2018: 211; Scharloth et al., 2020; Stumpf and Römer, 2018). exposure/revelation was added as one category, as they are not mutually exclusive and share a significant fraction of terms. Third, an accumulation of markers of distance (i.e. qualification through adjectives such as ‘so-called’, or the use of quotation marks around concepts that are rejected) is considered indicative of conspiracy discourse (Keilholz and Obert, 2018: 215; Stumpf and Römer, 2018).

Furthermore, a separate category conspiracy contains metaphors prevalent in conspiracy discourse. Butter identified imagery for the demonization of conspirators. In such narratives, the unknowing person is often described as a puppet being played by the conspirators. Allusions to the imagery of the theater and ‘taking a look behind the curtain’ are rife. The imagery of military action (for conspiracies from the outside) or of infection (for conspiracies from the inside) for the spread and advancement of the conspiracy is also very important, as well as the construction of the masses as blind, asleep or enslaved and the language of sexual abnormality for suspected groups of conspirators (Butter, 2020: 58–63).

Finally, a dimension that is not only conceptually related to conspiracist thought but is arguably situated at the structural core of most conspiracy theories is antisemitism (there are multiple studies dealing extensively with the subject, e.g. Byford, 2011; Cohn, 1998; Hammel, 2018; Imhoff, 2020; Waibl-Stockner, 2009). One reason for this is the importance conspiracy theories have had for antisemitism over various periods in history, with changing functions of anti-Jewish sentiments. ‘The representation of the Jew as an evil and disruptive figure, equipped with almost unlimited power, has been a recurrent feature of both premodern, religious anti-Judaism and modern nationalist and racist antisemitism’. (Simonsen, 2020: 357). Most of the characteristics of conspiracy discourse discussed so far can likewise be applied to antisemitic stereotypes. Jews, perceived as a homogeneous and globally connected group are thus well-suited for playing a major role in large-scale conspiracy imaginations. For all the premodern elements of conspiracist antisemitism pervading until today, the original rootedness in Christianity was replaced by a sense of Jews endangering the nation state, antisemitism thus becoming ‘a hallmark of the conservative and nationalist right’ in the 19th century (Simonsen, 2020: 360). It is at this point that the notion of a ‘Jewish world conspiracy’ is developed and promoted by anti-revolutionary movements and linked either to a growing influence of capitalism, or to the spread of communism. The Shoah has proved breeding ground for a further twist in antisemitic conspiracism, namely the denial of the murder of six million Jewish people within far-right movements. Holocaust denial is entirely based on conspiracy theories, the argumentation of which is reconstructed by Simonsen (2020: 365), culminating in the assumed Jewish ‘plot’ to gain support for the state of Israel.

Many of the elements of conspiracy discourse discussed in this section are also subjects of populism research. How exactly the two concepts are related and what this means for an analysis of conspiracy discourse in the context of right-wing populist discourse in Germany is examined in the following section.

### Linking conspiracy discourse and right-wing populism

The questions and issues discussed in research on conspiracy theories overlap substantially with those discussed in populism research, which justifies further interrogation of the connection between the two phenomena (Bergmann and Butter, 2020: 332–333). Populism is studied in a variety of disciplinary and interdisciplinary approaches. For all their differences in how they define populism exactly, three core elements can be identified throughout the research: (1) the reference to ‘the people’, (2) anti-elite sentiments and (3) a conception of ‘the people’ as homogeneous, monolithic, with strong exclusion strategies toward ‘Others’ or ‘the Other’ (Woods, 2014: 3). Expressed in terms of an ideational approach, populism can be defined as a ‘*thin-centered ideology that considers society to be ultimately separated into two homogeneous and antagonistic camps, ‘the pure people’* versus *‘the corrupt elite’, and which argues that politics should be an expression of the* volonté générale *(general will) of the people*’ (Mudde and Rovira Kaltwasser, 2017: 6 [italics in the original]). This ‘thin-centered’ ideational core consequently links populism to other – ‘thick-centered’ – ideologies, such as fascism, nationalism or socialism (Mudde and Rovira Kaltwasser, 2017: 6). Moffit and Tormey conceive of populism as a ‘political style’ (2014) capturing this ideational core, which enables a language-based study of populist discursive practices corresponding to the study of conspiracy discourse outlined above, as ‘style and substance are thus interlinked in populist politics’ (Bergmann and Butter, 2020: 332).

However, even as research on conspiracy theories often mentions their possible function for populist or authoritarian politics and vice versa, there is a lack of research studying the connection explicitly. Bergmann (2018) establishes that, while conspiracy theories ‘can be tailored to any political view’ and populism can take different forms, ‘the two unite as an especially powerful force within the field of the nationalist far-right’. (Bergmann, 2018: 105; see also Byford, 2011; Priester, 2012; Dreesen, 2019). As Wodak explains with a focus on the instrumentalization of fears: ‘*Conspiracies* by enemies within and outside the nation are part and parcel of the discursive construction of fear by far-right populists’. (Wodak, 2021: 94 [italics in the original]).

Focusing on RPC discourse thus calls for an expansion of the category system detailed above. The following elements are neither exclusive to right-wing populist discourse nor to conspiracy discourse, but relevant dimensions for conspiracy discourse within right-wing populist contexts. Because of its ‘thin-centeredness’, populism is adaptable to various subjects. However, the relatively stable core elements of populism (anti-elitism, appeal to a monolithic ‘people’ by simultaneous exclusion of ‘Others’) can arguably be found in a lot of opportunistic right-wing slogans of recent times, such as ‘Klimahysterie’, ‘Ökoterror’, ‘Gendergaga’ or, as of late, ‘Corona-Diktatur’. While not depicting any of these topics specifically nor ‘populism’ in general, the categories of RPC-Lex are designed to illuminate a certain kind of populist discourse, namely at the intersection of right-wing populist and conspiracy thinking. Therefore, most categories have relevance even for the study of RPC discourses concerning changing topics not explicitly included in the conception of RPC-Lex.

While anti-elitism has been established as a cornerstone of both conspiracy discourse and right-wing populism, right-wing populist conspiracy theories add a nationalist dimension to the concept. This nationalism is founded on the assumption of an ‘external threat to the nation’, which the country’s elite is not hindering or even ‘siding with external forces’, necessitating the protection of ‘the people’ and, by extension, the nation against the threat (Bergmann, 2018: 12). Within this construction of an external threat – through processes of Othering – lies the reasoning behind anti-immigration and islamophobic rhetoric in RPC discourse (Bergmann and Butter, 2020: 331–332). Its most extreme and best-known manifestation is found in the superconspiracy theory of the ‘Great Replacement’, which states the belief in a ‘white genocide’, orchestrated by Islamic countries in collaboration with Western elites. The influence of such conspiracy theories is reflected by violent terrorist attacks carried out in the name of preventing this ‘Great Replacement’ (Anders Breivik’s attacks in Oslo and Utøya, 2011, the El Paso shooting 2019, the Christchurch mosque shooting 2019, to name a few).

An essential dimension of such RPC narratives is the topos of apocalypse/downfall, (Priester 2012: 42). The always impending yet never quite arriving apocalypse is fed as ‘one major strategy of its [i.e. the far right’s] actors’, as they ‘address and inflame fears in order to justify extraordinary political approaches’ (Fielitz and Marcks, 2019: 5). The initially vague threat to the nation thus undergoes (largely rhetorical, Bergmann and Butter, 2020: 332) radicalization and can be expanded into entire ‘extinction narratives’ (Fielitz and Marcks, 2019: 6; see also Wodak, 2021: 253). The rhetoric of scandalization Scharloth (2017) has been made out as characteristic of the right-wing populist ‘style’ of the party *Alternative for Germany* (see below). Thus, conflict is emphasized in order to engage readers and polemicize complex subjects, a rhetorical strategy of conspiracist language use, too (Keilholz and Obert, 2018: 211). Terms that fall into this category are also common in tabloid journalism and linked to sensationalism, outrage and the economics of online publishing (‘clickbait’). Closely linked to fears of extinction and to the agonal constitution of ‘the people’ versus internal (‘the elites’) or external (‘the Other’/the immigrant) threats is the need and call for protest and rebellion. Wodak calls it ‘The “good” fight’ that justifies the ‘right to defend ourselves against “the others”’ (2021: 9). The call to protest and rebel against the corrupt system follows the argument that the nation and its people need protection in order to prevent their destruction and resolution, that is, the apocalypse (Fielitz and Marcks, 2019: 6).

In addition to these interconnected categories, anti-genderism/anti-feminism is an integral part of both conspiracy and right-wing populist discourse. Feminism and the ‘gender-agenda’ are often construed as conspiracies themselves, aimed at destroying the nuclear family, traditional conservative values and/or masculinity and femininity in general, in addition to ruining children through sexual education at school thought to sexualize them early on (Näser-Lather, 2020; Thiem, 2020). This dimension is shared by right-wing populist agendas (see e.g. Dietze and Roth, 2020). At the same time, right-wing populist rhetoric incorporates seemingly paradoxical views on women’s rights: Concerning Muslim women, they stand for the protection of ‘liberal values’, concerning white women, they stand for reactionary reproductive rights and conservative ideals of motherhood (to child and nation) (Farris, 2017; Wodak, 2021: 27–28).

Finally, there is another conglomerate worth investigating as a category of RPC discourse, namely esotericism. There is, first, the nexus between esotericism and the fascist Aryan ideology of the Nazis, claiming Aryan/Germanic people to be the true ‘*Übermensch’* in the divine order of the world. Especially for the German context, a lot of research has gone into the nature of the relationship between esotericism and fascism (Freund, 1995; Gugenberger and Schweidlenka, 1987; Heller and Maegerle, 2001; Kingsepp, 2015; Schweidlenka and Gugenberger, n.d.; Staudenmaier, 2010; Strube, 2012). However, esotericism can be found within other conspiracy beliefs, not necessarily directly or at least not consciously linked to a right-wing, let alone a fascist, agenda. Thus, not only esoteric terminology with fascist undertones is relevant, but also terminology pertaining to conspiracist beliefs about the corrupt intentions of conventional medicine (including vaccines), the dangers of new mobile communication standards (i.e. 4G, 5G) or about the non-existence of climate change. Most esoteric conspiracist beliefs are fueled by mistrust of the establishment and the search for an alternative truth that is being kept from ‘the people’.

These theoretical considerations show the strong structural connection between conspiracy and right-wing populist discourse. Combining the two terminologically into RPC discourse acknowledges these overlaps and allows to study interrelated elements of both discourses. While neither conspiracy nor right-wing populist language consist of absolutely fixed linguistic elements, relatively stable features of both discourses can be discerned (see Table 1 for an overview of all RPC-Lex categories introduced above, grouped according to the three dimensions ‘style’, ‘antagonists’ and ‘topoi’). Precisely because the ‘particular combination’ of these ‘psychological, structural and functional qualities […] can vary greatly according to context’ (Butter and Knight, 2020: 3), observing such combinations of conspiracy discourse elements in their intersection with elements of right-wing populist discourse constitutes the main concern of this paper and the focus of the RPC-Lex dictionary. Right-wing populism does not necessitate a belief in conspiracy theories or the participation in their spread, and vice versa. However, through their structural similarities, synergy effects can aid a broader support for movements that would ordinarily find themselves at the fringes of social and political discourse.

### The evolution of right-wing populism in Germany

‘There is no threat to Western democracies today comparable to the rise of right-wing populism’, writes Mackert (2019: 1). While the rise of right-wing populism has been observed since the 1990s at least, more recent developments confirm its advance, usually linked to a profound sense of disenchantment with the democratic processes and institutions perceived as having failed and alienated ‘the people’ (Vorländer et al., 2018: 169). This is often illustrated by such issues as the Brexit vote, the election of Donald Trump or election successes of right-wing populist and extreme right or authoritarian parties across Europe, for example in France, Austria, Germany, Poland, Hungary and Italy. Germany as ‘Europe’s most influential member state’ (Lees, 2018: 299) holds an especially critical place on the European political plane. To exemplify the strengthening of right-wing populism in Germany, it is helpful to consider the two largest movements of recent years, namely the political party *Alternative for Germany* (AfD) and the association of ‘Patriotic Europeans against the Islamization of the Occident’ (Pegida: ‘Patriotische Europäer gegen die Islamisierung des Abendlandes’).

Pegida was founded in 2014 in the East German town of Dresden, state capital of Saxony. First only a private Facebook group, the group soon organized regular ‘demonstration walks’ through Dresden city on Mondays. The ‘refugee crisis’ of 2015 brought Pegida renewed publicity and the movement gained momentum in other parts of Germany (Vorländer et al., 2018: 2–5). Its conspiracist core is suggested in the name of the movement, namely the fear of the ‘Great Replacement’ through uncontrolled immigration and Islamization. As the name of the group clearly states, the main conditions for belonging to ‘the people’ are ethnicity and religion. This right-wing ideology is supported by the chanting of ‘lying press’ (‘Lügenpresse’) at Pegida demonstrations (Nachtwey, 2016: 135). Typical for the new forms of right-wing populism is the suggestion that there is no real freedom of speech and of opinion because ‘the establishment’ is supposedly forcing its agenda on citizens through the ‘system press,’ hence necessitating civil protest (Gadinger, 2019: 130). Pegida thus combines Islamophobic and anti-immigrant fears with a general anti-elitism including the ‘mainstream media’ (Dostal, 2015: 523).

While Pegida remains a non-parliamentary movement, which means it can employ a more radical anti-democratic rhetoric, the AfD, founded in 2013, took the parliamentary route in their efforts to challenge the political system. Some argue that it can be viewed as the parliamentary arm of the Pegida movement (Dostal, 2015: 523). Following their success at the 2017 Federal election, the AfD became the first right-wing party that made it to the German and European parliament since the 1950s, even becoming the third largest party grouping in the German *Bundestag* (Lees, 2018: 295–296; see also, for the study of the AfD’s right-wing populism Häusler, 2016; Wildt, 2017). Similarly to Pegida, social media presence plays an important role in the AfDs success and presence (Serrano et al., 2019). The self-positioning of the AfD as a protest party constitutes a major pull factor for supporters who are disillusioned by a political system they do no longer perceive as representing, let alone benefiting them, thus a political system lacking legitimacy, finding themselves in a crisis of representation (Nachtwey and Heumann, 2019: 439). In turn, the vote for the AfD is itself perceived as an act of protest against a corrupted system (Nachtwey and Heumann, 2019: 451).

To summarize, there has been a considerable growth of right-wing populist protests, movements and parties in Germany, which substantially base their *raison d’être* on conspiracy theories such as the ‘Great Replacement’ while making vast use of online communication and social media (Puschmann et al., 2020). Germany is one of the most influential political and economic players in Europe and is home to a stack of right-wing (populist) movements that have gained momentum over the course of the past decades. A more detailed understanding of how right-wing populist ideas feed off and advance conspiracy thinking in the German context is not only interesting from a communication scientific viewpoint, but is also politically relevant. In order to study this development in more detail, we first constructed and then validated our computational dictionary, a process that we describe in detail in the following section. While focusing on German content only, studying different corpora (Facebook data and alternative news media articles) provides a cross-platform perspective within which we apply our conceptual frame of RPC discourse.

## Dictionary construction and validation

This paper employs two sets of methods for dictionary design drawn from the toolkit of the text-as-data approach increasingly popular in media and communication research as well as neighboring fields such as political science and sociology (Boumans and Trilling, 2016; Grimmer and Stewart, 2013; Maerz and Puschmann, 2020; Welbers et al., 2017). The first set of methods is used to create the dictionary and combines theoretical considerations and manual annotation with automated procedures for expanding, cleaning and optimizing the dictionary. The second set of tools is used to apply the dictionary and interpret the results of this application in order to demonstrate the dictionary’s usefulness for research purposes.

Based on the quanteda package (Benoit et al., 2018), the main application of a dictionary is to count the number of words in each message (or alternatively the entire corpus) that are also contained in a specific dictionary category. Relative shares of each dictionary category can thus be calculated, either on the basis of overall words in a corpus or by assigning each text (or paragraph or sentence) in the corpus a label based on which category of the dictionary receives the most hits. These shares can then be compared along an additional variable – typically source, author, political leaning on one side, or time on another – to identify differences and gauge shifts over time. Finally, the results can be subjected to both statistical tests and used as a model input, for example in regression analysis (Welbers et al., 2017).

Computational dictionaries represent one – today quite traditional – set of tools to study textual data quantitatively (Young and Soroka, 2012). While their ease of use represents an advantage, dictionaries have been criticized for being less adaptable to different types of data and for a lower reliability over newer approaches, particularly supervised machine learning, especially when they are imperfectly adjusted to the data at hand or when the aim is to assign texts discrete categories, as is the case in standardized content analysis (Chan et al., 2021; González-Bailón and Paltoglou, 2015). The significant limitations that apply when comparing dictionary performance to human coding combined with supervised machine learning necessitate stringent validation of any dictionary before application. Baseline validation of RPC-Lex was carried out via human labeling of sentences against the codes assigned by the dictionary based on majority voting with all limitations that this approach engenders (Barberá et al., 2021). While the accompanying release of the dictionary has provisions to make the use as a classifier feasible in principle, we caution users to carefully inspect the material they apply the dictionary to in order to safeguard against errors and enclose performance metrics to enable users to make informed choices when applying the dictionary to their own material (see Chan et al., 2021; Song, 2020), for suggestions on how to validate results in such scenarios).

### Construction

**Figure 1.**
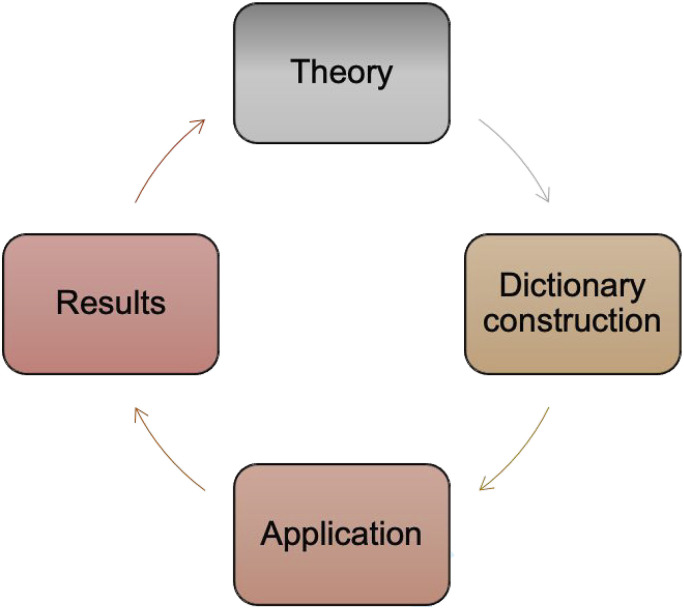
Methodological loop in dictionary construction and application.

There is no single accepted method to construct computational dictionaries. As dictionary analyses are ‘more deductive in nature and presuppose very detailed domain knowledge’ (Maerz and Puschmann, 2020: 44), the RPC-Lex dictionary categories as well as the terms chosen to populate them were developed based on the theoretical foundation illustrated above. In this way, the terms chosen for the dictionary can be understood as indicators for the theoretical concepts they are supposed to measure (Gründl, 2020: 6). At various stages of the process, inductive and explorative loops were performed based on the material described below (see Figure 1).

In an undergraduate research seminar on conspiracy theories taught in the fall of 2019, students were asked to compile word lists on the basis of theoretical texts (Butter, 2018; Detering, 2019; Horn and Rabinbach, 2008; Hunger, 2016; Krause, 2011; Melley, 2000; Strässle, 2019), individual international and German-language case studies (*The Protocols of the Elders of Zion*, Compact-Magazin, KenFM, Alex Jones, Daniele Ganser, Eva Hermann) and research on (media) events occurring in the period of time our corpora cover (i.e. the ‘refugee crisis’ or the ‘Cologne New Year’s Eve, 2015/2016’). The result was an uncategorized collection of 1512 entries (1315 unigrams, 197 n-grams) and a list of abstract language features (e.g. quotation marks).

In order to arrive at a dictionary that is extensive enough to provide a good *recall* yet exact enough to be *precise* (Gründl, 2020: 6), the authors manually cleaned and expanded this first word list, and arranged it into categories in an extensive iterative process. First, a literature review on both conspiracy discourse and right-wing populism was conducted. The deductive category framework derived from these theoretical considerations passed through different stages, re-evaluating the suitability and applicability of categories for the corpora described below. Second, while the rationale behind individual categories is outlined in the previous sections, literature was also consulted to expand the initial word list and thus to populate the categories.

For the linguistic category of scandalization, following Scharloth (2017), verbs of conflict (Dornseiff, 2004; Harras et al., 2004; Scharloth, 2017), intensifiers (Scharloth, 2017) and terms with negative and derogatory connotations (Dornseiff, 2004; Scharloth, 2017) were gathered. For suspicion/manipulation, exposure/revelation and markers of distance, terms were extracted from the studies dealing with their importance for conspiracist (Keilholz and Obert, 2018; Stumpf and Römer, 2018; Scharloth et al., 2020) and right-wing populist (Ebling et al., 2013; Römer and Stumpf, 2019; Scharloth, 2017) discourse. In addition, sociological studies conducted to measure conspiracy belief and right-wing populism in Germany were also taken into consideration, alongside newspaper articles reporting on the results of these studies (Decker and Brähler, 2018, 2020; Rossteutscher et al., 2019).

The category of conspiracy was populated with the metaphoric imagery identified by (Butter, 2018: 93–100) , as well as with common terms pertaining to specific, wide-spread conspiracy theories (about 9/11, vaccinations, *Reichsbürger*, UFOs) or to conspiracy theories especially relevant for our data (Holocaust denial, the Great Replacement, the New World Order). Other categories of the dictionary are essential to conspiracist rhetoric, too, but have been operationalized separately for better comparability of the overlaps of conspiracist and right-wing populist discourse. This concerns, for example, a vocabulary of exposure/revelation or of suspicion/manipulation. The category of antisemitism was populated on the basis of studies of antisemitic language (Schwarz-Friesel and Reinharz, 2012; Siehr and Seidel, 2009) and other relevant literature (Blumesberger, 2009; Bolaffi et al., 2003; Fischel, 2020; Hammel, 2018; Suermann, 2019), as well as through explorative web searches.

anti-elitism includes terms denoting a hostility toward the political establishment, toward science and scientific institutions and toward the ‘mainstream media’. Terms were gathered based on the linguistic research defining anti-elitism as a relevant element of conspiracist and or right-wing populist discourses (Keilholz and Obert, 2018; Schäfer, 2018) and expanded by terms referring to specific political actors and parties relevant to the German context. Numerous terms for the category of apocalypse/downfall were already included in the initial word list. Additional terms were included from Horn (2014), Wodak (2021), Weiß (2017), as well as Fielitz and Marcks (2019).

The category of nationalism (ger) gained terms from various explorative web searches and relevant literature (Häusler, 2019; Niehr and Reissen-Kosch, 2018; Steinke, 2019; Wodak, 2018). While the nationalist vocabulary was designed to mark patriotic sentiments about Germany, the anti-immigration/islamophobia list is closely linked to the former and often derived from the same literature and websites yet focusses on this one specific perceived threat to the nation. Within the right-wing populist context of our corpora, terms populating the anti-gender/anti-feminism category are linked to an anti-immigration rhetoric in the broader sense, including homophobic vocabulary of a ‘feminized’ society no longer able to keep the ‘fatherland’ pure from outside influences. The category was populated based on literature (Dietze, 2019; Dietze and Roth, 2020; Höcker et al., 2020; Jäger et al., 2019; Näser-Lather, 2020; Sauer, 2017; Strube et al., 2021; Wamper, 2019).

Taking into consideration the difficulties of clearly defining esotericism for RPC discourses, terms for this category were chosen based on literature dealing with the specific intersection of esotericism and right-wing thought (Freund, 1995; Gugenberger and Schweidlenka, 1987; Heller and Maegerle, 2001, 2007; Kingsepp, 2015; Staudenmaier, 2010; Strube, 2012) as well as specific esoteric fields currently popular, such as UFOs, shamanism, alternative medicine, eugenics, angels and demons, or mobile communication standards.

Following the development of an initial seed dictionary based on this review of the relevant literature, we next calculated the occurrences of the seed terms in a reference corpus consisting of comments in German-language right-wing Facebook groups, particularly those associated with Pegida and the AfD, spanning the period from 1 January 2015 to 24 May 2016 (see Puschmann et al., 2020, for details on this corpus). Co-occurrence and KWIC (keyword-in-context) searches as well as spot checks on qualitative samples for all categories were carried out using the reference corpus. We also relied on computational word similarity metrics to identify terms that occurred in conjunction with our seed terms in the reference corpus. In addition, further websites spreading conspiratorial and/or right-wing content were searched (e.g. the sites *WikiMANNia *and* Metapedia*, both modeled after Wikipedia). This latter step delivered a number of codes, relevant predominantly to the categories of conspiracy and antisemitism. Examples include numbers (e.g. 18 and 88, the letter number codes for ‘Adolf Hitler’ and ‘Heil Hitler’, respectively), acronyms (e.g. ‘ajab’, analogous to the anti-police slogan ‘acab’), portmanteaus (e.g. ‘jewnited states’, ‘USrael’, ‘germoney’) or contractions (e.g. ‘jdn’ for ‘Juden’, ‘zckn’ for ‘Zecken’). However, these coded messages need constant updating, considering how quickly and creatively online communication can react to censorship or uncovering of codes.

Finally, for all categories synonyms of verbs, nouns and adjectives were generated using the Wortschatz Leipzig online resource, Dornseiff’s *Der deutsche Wortschatz nach Sachgruppen* (2004), Harras’ et al. *Handbuch deutscher Kommunikationsverben* (2004) and the online resource of the DWDS. Asterisks were used in a first automated step to gather all relevant variants within the corpora, and then expanded to identify a large number of additional derivative forms that were added to the dictionary. For a German language dictionary especially, using regular expressions to capture the most used grammatical variations returns higher precision than using asterisks or stemming (Gründl, 2020: 8).

The enriched dictionary was once again cleaned manually, removing most function words, highly polysemic and high-frequency nouns, adjectives and verbs as well as terms occurring in more than three categories. Unclear terms, URLs, hashtags and other faulty entries were removed. Further flection forms were added where necessary, to arrive at a wildcard-free dictionary. The result was a global dictionary with 14,105 entries (including those occurring in multiple categories). To aid the process of validation, the categorization and relevance of terms was once more critically evaluated by all authors. The dictionary was thus reduced once more following the same multi-person procedure to ensure accuracy and checked against new and relevant publications to ensure a high recall. The final dictionary consists of 10,829 unique entries, distributed over 13 categories.

A complicating factor that arises when applying computational dictionaries is that outcomes are influenced by baseline word frequency in a language, that is, certain words included in the dictionary may be far more likely to occur than others, and if the distribution of such words differs between categories this could adversely influence results (see e.g. Rauh, 2018). To provide potential users of the dictionary with a resource to counter this problem, an additional step toward enrichment was taken. We matched the terms contained within RPC-Lex with the DeReKo reference corpus for German (Kupietz et al., 2018), specifically with the most recent release of the DeReWo frequency-annotated word list, which provides valid base frequencies of the 100,000 most frequent terms in German. This is helpful because it allows users of RPC-Lex to weigh the occurrence of terms in their data against an expected base frequency to judge how indicative they are of a specific topic. For example, a single occurrence of the term *Polizeistaat* (police state; base frequency of 3760) can be regarded as more indicative of RPC than an occurrence of *Freiheit* (freedom; base frequency of 466,307). By applying the base frequency as a weighting factor or excluding highly frequent words entirely when applying the dictionary, users are able to further improve the validity of their approach.

### Validation

Before formal validation of the dictionary was undertaken, we conducted a comparison with another dictionary recently developed for the study of right-wing populism by Gründl (2020) in order to characterize the degree to which the two resources describe similar concepts. This step does *not* represent a validation, but instead allows dictionary users to better evaluate the relative suitability of both resources to particular research questions they may be interested in. It should be pointed out that we did not *base* our dictionary on Gründl (2020), which was published when dictionary construction of RPC-Lex was well under way. Our aim was to determine whether substantial overlap exists between the terms incorporated into the two dictionaries, particularly for those categories assumed to be strongly influenced by right-wing populist concepts, rather than those categories in our dictionary that go beyond the categories captured in the Gründl dictionary. We achieved this by calculating the percentage share of terms in RPC-Lex also contained in Gründl (2020), differentiating by category. This results in an overlap of terms between the two resources that ranges from 38% for anti-elitism to 10% for protest/rebellion. The range in overlap also illustrates the conceptual differences between the two dictionaries. Those categories with the highest degree of overlap exist in both dictionaries, while those with the least overlap are specific to our dictionary and therefore missing from Gründl’s. The latter are categories crucial to the larger concept of RPC discourse outlined above. Overall, the similarity of the two dictionaries is quite strong, particularly for the categories in RPC-Lex that Gründl also identifies. While this step does not represent a validation, the overlap serves to demonstrate the degree of similarity arrived at via two independent theory-driven approaches.

Following this preliminary step, we compared the classification of texts via the dictionary to the judgement of human coders. Two student assistants were first provided with a basic code book describing the 13 categories in the dictionary along with a set of anchor example sentences (see our OSF repository for further details). An in-depth discussion of the examples among the coders and two of the authors was also conducted to clear up open questions on the composition of the categories. No in-depth coder training or formal pretest was conducted in order to determine the reliability with which the categories could be consistently coded with only minimal instructions. In the next step, the two coders independently labeled 2,500 sentences randomly sampled from a corpus of comments posted to 25 RPC Facebook pages using the dictionary categories as labels. Of these, 2,494 could be retained for further analysis, with six discarded due to technical error. The coders achieved agreement in independently selecting the same category in only 50% of coded sentences, with considerable variation between categories (see Online Appendix 1). The 1,251 consensus cases where both coders chose the same category were very unevenly distributed between the 13 categories, with some categories occurring only a few times in the data. The appendix contains an overview of the consensus categories’ distribution.

The dictionary was then used to independently label the sentences for which consensus was achieved. This was realized by simple word matching based on the categories, with the label associated with each sentence being the category that had received the most hits. Only those sentences with three or more matching words were labeled, with the rest assigned the ‘NA’ category. We then proceeded to calculate agreement between human consensus and dictionary-based labeling. The dictionary-based labeling achieved an accuracy of 0.74 (95% CI: 0.72, 0.77, no information rate: 0.3173) with some variation between categories. Due to the very small number of observations in some of the categories, reliability of the dictionary-based labeling for these categories should not be presumed. However, even these categories should be useful to RPC scholars provided they are carefully validated on the data under study and assuming the data is sufficiently similar to the RPC style previously described. Table 2 shows benchmark performance per category. The appendix also contains further information on the training material, the category distribution within the material, the level of agreement between the two human coders, as well as a confusion matrix.

**Table 2.** Model coefficients per dictionary category.

	Sensitivity	Specificity	Pos pred value	Neg pred value	Precision	Recall	F1	Prevalence	Detection rate	Detection prevalence	Balanced accuracy
Scandalization	0.93	0.98	0.65	1	0.65	0.93	0.77	0.04	0.04	0.06	0.95
Suspicion/Manipulation	1	0.97	0.57	1	0.57	1	0.73	0.04	0.04	0.06	0.99
Exposure/Revelation	0.96	0.99	0.63	1	0.63	0.96	0.76	0.02	0.02	0.03	0.97
Markers of distance	1	1	0.85	1	0.85	1	0.92	0.01	0.01	0.01	1
Antisemitism	1	1	1	1	1	1	1	0.01	0.01	0.01	1
Anti-elitism	0.74	0.9	0.78	0.88	0.78	0.74	0.76	0.32	0.24	0.3	0.82
Anti-immigration/Islamophobia	0.67	0.96	0.86	0.89	0.86	0.67	0.76	0.26	0.18	0.2	0.82
Anti-gender/Anti-feminsim	0.67	1	1	1	1	0.67	0.8	0	0	0	0.83
Conspiracy	0.82	0.97	0.58	0.99	0.58	0.82	0.68	0.05	0.04	0.07	0.89
Apocalypse/Downfall	0.92	1	1	1	1	0.92	0.96	0.02	0.02	0.02	0.96
Protest/Rebellion	0.94	0.99	0.79	1	0.79	0.94	0.86	0.04	0.04	0.05	0.96
Nationalism (GER)	0.97	0.99	0.75	1	0.75	0.97	0.85	0.03	0.03	0.04	0.98
Esotericism	1	1	1	1	1	1	1	0	0	0	1
NA	0.56	0.94	0.64	0.92	0.64	0.56	0.6	0.16	0.09	0.14	0.75

It should be noted that this approach is hardly suitable to distinguish between RPC and non-RPC content as a result of the broad coverage of the dictionary. Using the dictionary to classify texts unequivocally requires human validation of a random sample of texts, including cases which do not match any category (see Chan et al., 2021). It is also worth pointing out that the dictionary was developed in a theory-led process, rather than having been explicitly designed for the material that we then applied it to in this step, with the validation tentatively suggesting that using it to choose among categories may yield satisfactory results under the right circumstances. However, the uneven category distribution is a considerable limitation, with the categories antisemitism, anti-gender/anti-feminism and esotericism too sparse in the training data to be considered validated (*n* < 20).

Having taken these steps to safeguard the validity of the computational resource, we provide an application of the RPC-Lex dictionary to online discourse at the intersection of right-wing populism and conspiracy theory in the following section.

## Application to two use cases

In the following, we conduct an analysis that relies on two different large textual datasets, described in more detail in this section, which total 2.4 million documents and approximately 164 million tokens (see Table 3). Both corpora are predominantly German-language, though some content in other languages may show up particularly in social media sources. While the data contained in the Alternative News corpus is in principle public (though restrictions to sharing of raw data apply because of copyright), the data contained in the Facebook corpus was collected as part of a covert investigative journalism projection coordinated by German public service broadcaster BR and is subject to a usage agreement that explicitly forbids the authors from sharing the data in order to insure user privacy and shield the broadcaster from litigation.^3^

**Table 3.** Overview of corpora used in analysis.

Corpus	Period covered	Documents	Tokens
Alternative news corpus	January 2017–December 2019	123,581	95,638,059
Facebook corpus	May 2010–December 2019	2,286,607	68,414,806

The Alternative News data were collected in 2020 and 2021 and covers the period from 2017 to 2019. In the case of the Facebook dataset, a somewhat longer period, from 2012 to 2019, is covered. Both the Facebook and Alternative News datasets were collected via web scraping. Both corpora encompass discourses that are thematically related to the RPC narratives that form the conceptual core of the dictionary – in other words, they contain communication that should be picked up by the dictionary as relevant to the issue under study.

### Alternative news corpus

We applied the RPC-Lex dictionary to a full text corpus of news items from nine alternative news outlets discussed in the research literature on alternative news and conspiracy theories. Our understanding of alternative news outlets is based on the typology of Holt et al. (2019) who describe online news sources that exhibit a politically radical editorial policy and frequently circumvent journalistic norms. We base our selection on recently published studies of alternative right-wing news that provided reasoned lists of popular and influential news outlets (Boberg et al., 2020; Frischlich et al., 2020; Heft et al., 2019). Our selection of sources was furthermore influenced by two additional aspects. First, a substantial visibility in terms of Facebook engagement data, collected for another project, a parameter that added sites to the list that are not necessarily widely read independent of Facebook usage. Second, outlets being actively listed by German domestic intelligence (*Bundesverfassungsschutz*) as a potential danger to democracy (i.e. associated with violent threats), which applied only to a subset of sources. This resulted in a total of nine outlets of varying reach and visibility. We then scraped all URLs obtained from a particular source that had been shared on Facebook between January 2017 and December 2019 and applied the RPC-Lex dictionary to the data.

**Figure 2.**
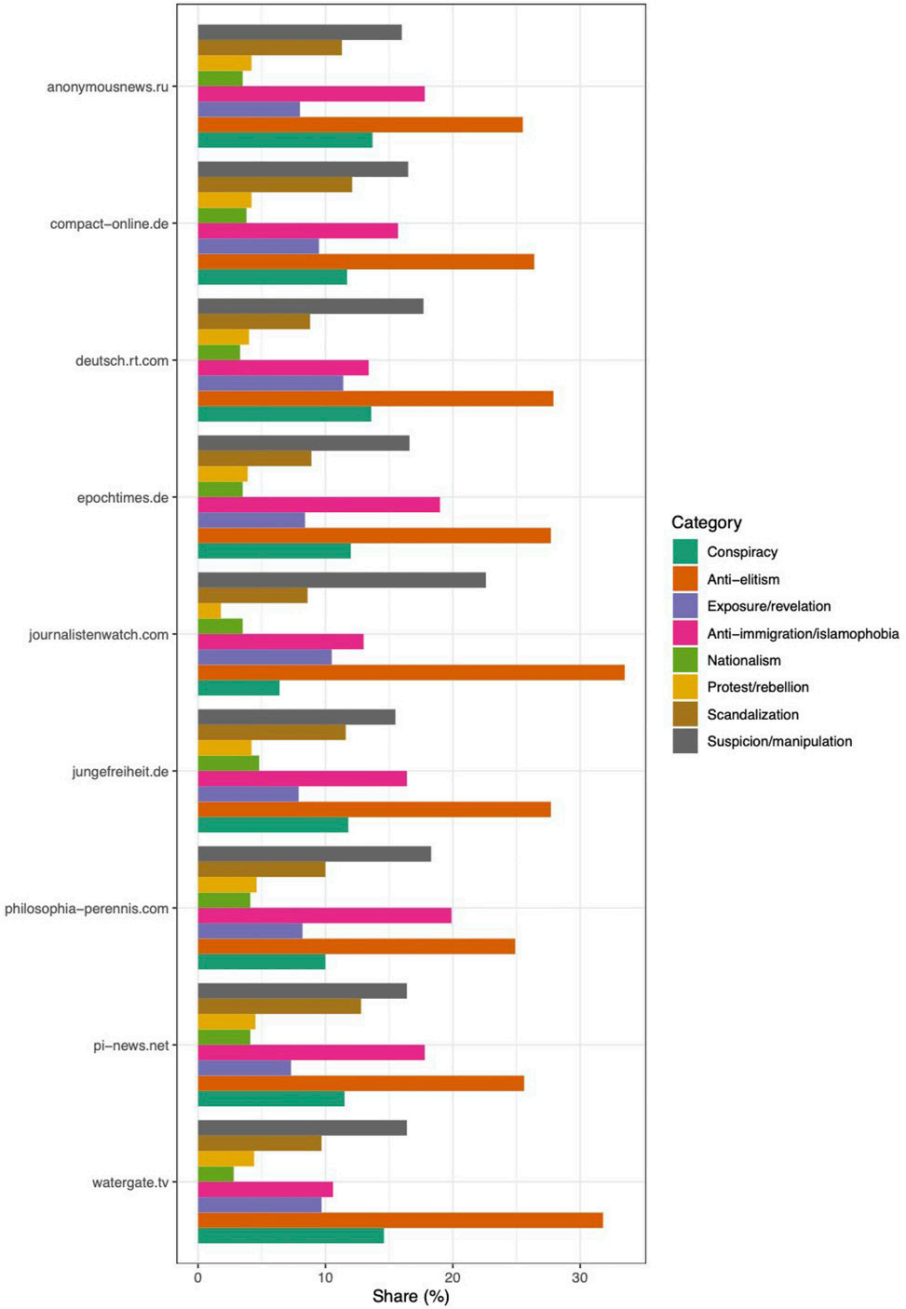
RPC-Lex categories in the Alternative News Corpus by source.

The result is a detailed profile of German right-wing alternative news sources covering specific aspects of RPC discourse, such as anti-immigration/islamophobia or nationalism. Figure 2 shows the distribution of the shares of eight of the 13 different dictionary categories among the nine different sources that we analyzed. The *X* axis shows the dictionary categories as well as the respective percentage share of sentences for which the category scored highest, while the *Y* axis shows the sources by name.

It is possible to characterize the sources in terms of their distributional characteristics in relation to particular clusters of categories, for example, sources that score high in exposure/revelation, protest/rebellion and conspiracy versus those that particularly emphasize the anti-immigration/islamophobia and nationalism categories. RT and Epoch Times tend to differ from traditional right-wing German news outlets such as Junge Freiheit and Compact in this respect. scandalization and anti-elitism are especially salient in Pi-News and Watergate TV. An outlier among the sources is Journalistenwatch which achieves the highest shares of any sources in the anti-elitism and suspicion/manipulation categories.

Crucially, such differences tend to align with differences in the type of news source, for example, RT comparatively deemphasizes anti-immigration/islamophobia and to a lesser extent nationalism, but leads in exposure/revelation and conspiracy, which hardly seems arbitrary considering its presumed strategic aims (Elswah and Howard, 2020; Yablokov, 2015). Sources that cater to different types of audiences thus become visible.

### Facebook corpus

The main aim of the application of RPC-Lex to the Facebook corpus is to show the ebb and flow of RPC discourse over time. The Facebook corpus was created as part of investigative reporting conducted by German public service broadcaster BR into the prevalence of right-wing hate speech in German-language Facebook groups. Reporters created fake Facebook profiles and thus gained access to a large number of non-public right-wing Facebook groups. Posts and comments from these groups were subsequently scraped, resulting in an archive that reaches back to 2010, though sample sizes were considered too small in the first several years for use in our analysis.

**Figure 3.**
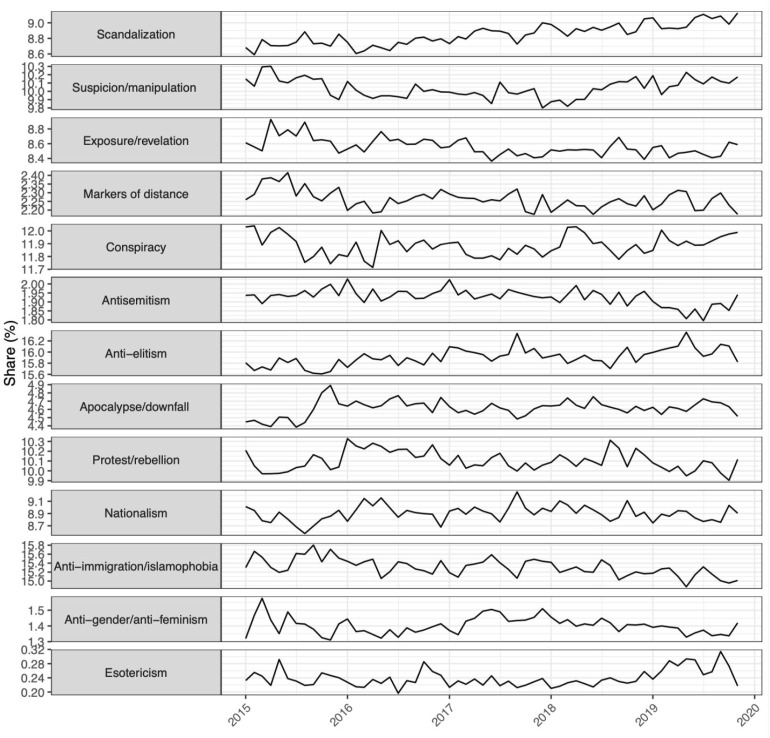
RPC-Lex categories in the Facebook corpus over time.

Figure 3 shows how the distribution of dictionary categories in the data changes over time in the period from 2015 to 2019. It is important to keep in mind that the basis is in this case a set of Facebook comments made by a large number of extremist right-wing Facebook groups over this time span. This contrasts with the Alternative News dataset in which the bases are news articles, which are generally longer and stylistically quite different from comments. As before, the variable of interest, in this case time, is shown on the *X* axis while the *Y* axis shows the 13 different categories and their percentage shares, here computed as number of terms matched with the respective category, rather than share of messages. As before, these shares differ considerably.

First, there is a set of categories that decreases over time. For example suspicion/manipulation, anti-immigration/islamophobia as well as antisemitism all decrease somewhat in the period from 2015 onwards, instead being superseded by other categories, though there is an uptick in antisemitism in 2019. scandalization increases in the period under study, though from a high base, as does anti-elitism. A second set of categories stays fairly stable distributionally over the period under study, e.g. nationalism, apocalypse/downfall and anti-gender/anti-feminism. esotericism is a special case in that it makes no net gains over most of the period, but is up in the time span since 2019, coinciding with the COVID-19 pandemic.

Some of these fluctuations match up with expectations more clearly than others. For example, the clear long-term increases in categories such as anti-elitism, anti-gender/anti-feminism or nationalism resonate with expectations more clearly than the decrease in certain categories, for example, conspiracy. There are clear reasons for this dynamic, however. For example, there is a strong correlation between current events and these fluctuation patterns. 2015 was the year of the so-called European ‘Refugee crisis’ and the Charlie Hebdo attacks in Paris. While there is clearly a link between these events and spikes in particular category shares (e.g. anti-immigration/islamophobia peaking in 2015), there are also long-term trends that seem independent of them, for example the overall salience of anti-elitism, anti-gender/anti-feminism and apocalypse/downfall. 2016 was the eventful year of Brexit, Donald Trump’s election as well as the terrorist attacks in Brussels, Nice, Berlin, München and Ansbach, leading to spikes in nationalism and anti-elitism. In 2017, there was a federal election and the initial failure to form a coalition government, coinciding with a rise in anti-elitism. Another notable event was the passing of legislature that legalized gay marriage in Germany, which was accompanied by a spike in anti-gender/anti-feminism rhetoric. This was followed in 2018 by an expansion in the 5G mobile network (spike in esotericism) and right-wing extremist riots in Chemnitz (spike in nationalism). Finally, 2019 saw a growth in the coverage of climate change as Germany experienced a heatwave (growth in apocalypse/downfall).

Perhaps, one of the most surprising developments is the lack of clearly visible growth in the conspiracy category. This points less to a true decline in conspiracy discourse than the evolution of the types of conspiracies in circulation. For example, conspiracies related to mobile communication standards were still a niche phenomenon in 2015. In contrast to most other categories in the dictionary, conspiracy presents itself as highly dynamic and a moving target, particularly when relying on a bag-of-words approach as our dictionary does.

## Discussion

In this paper, we have first described the concept of RPC discourse and then operationalized this concept by means of a computational dictionary. We have argued that (a) a meaningful nexus exists between conspiracy theory and right-wing populism and (b) that a computational dictionary represents a suitable resource for the comparative study of these mutually intertwined political phenomena. After having outlined the composition of our dictionary and taken steps toward validation, we have applied it to two large-scale corpora of online RPC content. This application has shown both the strengths and the weaknesses of our approach. In what follows, we will discuss first the limitations and then the potentials and future directions of the computational approach that we have presented.

Before we proceed in this direction, however, it is necessary to spell out why the concept of RPC discourse advances the field of political communication. As we have outlined, there exist significant affinities between right-wing populism and conspiracy theories that have been previously recognized but not (sufficiently) studied. A key reason for this mutual affinity lies in the antagonistic worldview articulated by right-wing populism. Powerful forces within and without a national political sphere are imagined to steer public opinion and make decisions to fundamentally alter society against the will of ‘the people’. These tendencies are clearly visible in the data we have analyzed for demonstration purposes. It also appears that certain types of media are closer to the style of alternative right-wing news than others (e.g. welt.de; cf. Puschmann et al., 2016). It is important to point out that the corpora do not fully cover the period of the COVID-19 pandemic, which is likely to be the explanation for the moderate growth of the conspiracy category in the diachronic Facebook data. We are also able to reliably detect patterns of increased interest in particular issues and the gradual fading of these patterns – for example, the so-called ‘refugee crisis’ of 2015–16 and its aftermath is clearly reflected in the data.

As is to be expected, there are also considerable limitations to our approach. These are partly related to the weaknesses of the bag-of-words approach in computational content analysis and partly a result of the conceptual difficulties of properly delineating different discrete categories describing discourse at the intersection of conspiracy theory and right-wing populism (Chan et al., 2021). While this problem arguably exists in computational content analysis generally and applies to any computational dictionary, it is exacerbated when the objects of study are as fluid and diverse as right-wing populism and conspiracy theory. Word embeddings in particular hold great promise in the context for improving computational dictionaries (Rodriguez and Spirling, 2022; Rudkowsky et al., 2018).

Crucially, RPC-Lex should be used only after careful validation as an RPC *classifier* and only on material that is considered RPC, rather than on a mix of RPC and non-RPC content. Our objective was to create a resource that distinguishes different styles, themes and antagonistic relationships *within* RPC discourse, rather than reliably determine whether a piece of content should be classified as RPC or not. This is partly based on our own assumptions regarding the use of the dictionary (to identify categories within RPC discourse) and partly due to the way in which the quanteda package, on which RPC-Lex is based, applies dictionaries.

It should also be noted that RPC-Lex does not measure ‘populism’, nor can its categories be defined as ‘populist topics’. As pointed out in the literature review, populism’s ‘thin-centeredness’ makes it opportunistic, adaptable to various subjects and lets right-wing populist actors invent new slogans or coded terms. Our focus is instead on relatively stable core elements of populism (anti-elitism, appeal to ‘the people’, exclusion of ‘others’) that make it possible to measure populist discourse independent of one specific topic. The usefulness of our dictionary to contexts other than the ones presented here must be critically evaluated. Dictionaries used to study policy areas such as security, environmental issues or healthcare can capitalize on a specialized lexis consisting of technical terms and jargon, use of which reliably signals the appearance of a certain category from within the dictionary – arguably an advantage over RPC. While the addition of topics into a dictionary like RPC-Lex follows inevitably difficult design decisions (Chan et al., 2021), we do claim a certain theoretical validity as safeguard against too severe contingency regarding our topic choices. This is achieved by a theoretically supported category conceptualization as a base for RPC-Lex. This is not to say that other topics are decidedly unfitting, or that our list of categories is exhaustive.

As we have described in the section on the composition of the dictionary, we have first qualitatively compiled a list of terms on the basis of the academic literature on right-wing populism and conspiracy theories and then drawn upon quantitative techniques to extend, revise and improve upon these seed terms. However, every corpus is different and, when applying the dictionary, it is of the utmost importance to carefully check the validity of the dictionary on the material under study via human coding, especially when the corpus is based on digital discourse such as social media or news texts. This is particularly important for categories such as conspiracy, which have a tendency to change rapidly as new phrases and keywords enter into the conceptual vocabulary. In addition, the coded messages included in RPC-Lex need regular updating, considering such a list can never be complete and due to the quick and creative changes in online communication to circumvent censorship. These are concrete limitations that apply specifically to the dictionary we have developed and only to specific categories, which are more likely to change and degrade over time.

Considering these drawbacks, it is important to point out the specific advantages of the dictionary-based approach. As we have sought to demonstrate in our application of the dictionary, a key benefit is the ability to contrast the relationship of different categories to each other, in other words, to identify how strongly different tendencies within RPC discourse are expressed. This is not an end in itself. Contrasting category distributions truly becomes interesting when introducing a covariable such as the source of a news item (or the political leaning of that source), the poster of a social media message or a point in time, because then distributional differences among these covariables become visible, revealing structural differences between them (Klein et al., 2019). When applying such an approach as we have sought to demonstrate, the strengths of the dictionary are maximized because the results of the analysis no longer depend on individual isolated cases. While humans excel at close reading and in the actual interpretation of a piece of text, the computer is able to identify large-scale distributional differences in discourse that a human would not recognize. Assuming that the categories are well operationalized, that they fit with the data and that assigning a category to a piece of discourse is in fact possible on the basis of word usage (which it is not in all cases), this technique is accurate, reliable and highly scalable.

In addition to the quantitative use that we have outlined, it is also possible to combine a computational dictionary with qualitative methods. The most direct way of doing this is by using the dictionary categories only to identify relevant pieces of text which are subsequently read by a human. The ability to disentangle relevant pieces of discourse from a large social media corpus in this fashion can be of great advantage.

In closing, we would like to make three suggestions regarding the future development of both this resource and similar ones to benefit the field of interdisciplinary research into conspiracy theories. First, it would be beneficial to apply the same conceptual structure as we have used to other languages and other political discourses, both for the results themselves but also to improve the category system and make it more generalizable. We are convinced that the outlined structure translates – with certain limitations – to other political and linguistic environments. Second, a dictionary on conspiracy theories such as ours should be updated on a regular basis in order to capture recent developments such as conspiracy theories surrounding the COVID-19 pandemic. Third, we see further potential in our explorative procedure of integrating qualitative approaches and close reading into the development of the dictionary, for example, by consulting sources such as books, magazines or web pages that are influential in communities invested into conspiracy theories.

## Supplemental Material

Supplemental Material - RPC-Lex: A dictionary to measure German right-wing populist conspiracy discourse online Click here for additional data file. Supplemental Material for RPC-Lex: A dictionary to measure German right-wing populist conspiracy discourse online by Cornelius Puschmann, Hevin Karakurt, Carolin Amlinger, Nicola Gess and Oliver Nachtwey in Convergence

## Notes

1. The dictionary in multiple formats as well as the code book for manual analysis and our full text validation material including per-item coder decisions are available via OSF: https://doi.org/10.17605/OSF.IO/S48CJ

2. It is important to note that this use of the terminology ‘conspiracy discourse’ is independent of its use in other publications, where it usually signifies the media or popular cultural discourse about and around conspiracy theories (Aistrope, 2013; Deutschmann, 2020; Deutschmann et al., 2020; Soukup, 2002).

3. See https://web.br.de/interaktiv/hassmaschine/ for details on the project (in German).
