# Supplementary material for: RPC-Lex: A dictionary to measure German right-wing populist conspiracy discourse online
Source: Convergence (Lond). 2022 Jun 21;28(4):1144–71. doi: 10.1177/13548565221109440 (PMC9515517; doi:10.1177/13548565221109440)

Appendix for RPC-Lex: A dictionary to measure the prevalence of German right-wing populist conspiracy discourse

This appendix contains further material related to the validation of the dictionary-based content analysis presented in the article. Table A1 provides a comparison (in the style of a confusion matrix) between the two coders, while Table A2 represents an actual confusion matrix, with the human consensus as ground truth and the dictionary label as prediction. Finally, Table A3 lists the distribution of categories withint the coded data. Additionally, detailed validation data is available through our OSF repository.

Note that we use category shorthand codes to preserve space (APOC=Apocalypse/downfall, ASEM=Antisemitism, CONS=Conspiracy, DIST=Markers of distance, ELIT=Anti-elitism, ESO=Esotericism, EXPO=Exposure/revelation, GEND=Anti-gender/anti-feminism, ISLA=Anti-immigration/islamophobia, NA=NA, NAT=Nationalism, PROT=Protest/rebellion, SCAN=Scandalization, SUSP=Suspicion/manipulation).

The material used for the manual coding of dictionary categories was taken from comments posted to the following German-language Facebook pages between 2010 and 2019: *Alternative für Deutschland AfD, Dr. Frauke Petry, JUNGE FREIHEIT, Beatrix von Storch, NPD - Die soziale Heimatpartei, KOPP Online, Netzfrauen, DIE REPUBLIKANER, COMPACT-Magazin, KenFM, RT Deutsch, The Epoch Times - Deutsch, PI-News, Sputnik Deutschland, Contra Magazin, Nachrichten, die andere weglassen - Gegenfrage.com, Denken macht frei, Pegida NRW, PEGIDA Nürnberg, BRAGIDA, PEGIDA Hamburg, ZUERST! – Deutsches Nachrichtenmagazin, Deutsche Wirtschafts Nachrichten, Politikversagen, Bürgerbewegung PRO DEUTSCHLAND*. The choice of pages was informed by the assumption that the different categories of the dictionary are well-represented within this sample.

Table A1: Agreement on coded sentences between two coders and between human consensus and dictionary.

	Coder A														
Coder B		APOC	ASEM	CONS	DIST	ELIT	ESO	EXPO	GEND	ISLA	NA	NAT	PROT	SCAN	SUSP
	APOC	24	0	2	0	8	0	5	0	31	6	3	5	7	1
	ASEM	0	8	0	0	1	0	0	0	1	3	0	0	3	0
	CONS	6	1	61	1	47	0	13	0	15	23	10	3	7	10
	DIST	1	0	1	11	10	0	0	0	12	19	1	0	2	2
	ELIT	1	0	8	3	397	0	7	0	41	40	10	8	30	14
	ESO	1	0	3	0	1	1	0	0	0	13	1	0	0	0

	EXPO	6	0	7	1	36	0	25	0	30	93	7	0	8	6
	GEND	0	0	0	0	0	0	0	6	2	2	0	0	0	0
	ISLA	1	1	4	2	10	0	0	1	328	20	2	3	0	1
	NA	1	0	1	0	12	0	0	0	10	205	3	1	3	0
	NAT	8	0	1	0	10	0	1	0	12	15	37	2	4	0
	PROT	0	0	2	0	10	0	1	0	15	25	2	48	10	0
	SCAN	5	0	5	0	66	0	4	2	59	159	2	4	55	0
	SUSP	0	0	14	0	26	0	14	0	7	34	2	1	6	45

Table A2: Agreement between human consensus and dictionary as coder.

		Dictionary as coder													
Human consensus		APOC	ASEM	CONS	DIST	ELIT	ESO	EXPO	GEND	ISLA	NA	NAT	PROT	SCAN	SUSP
	APOC	22	0	0	0	1	0	0	0	0	1	0	0	0	0
	ASEM	0	8	0	0	0	0	0	0	0	0	0	0	0	0
	CONS	0	0	50	0	2	0	1	0	2	2	0	0	2	2
	DIST	0	0	0	11	0	0	0	0	0	0	0	0	0	0
	ELIT	0	0	17	0	295	0	6	0	14	33	6	5	10	11
	ESO	0	0	0	0	0	1	0	0	0	0	0	0	0	0
	EXPO	0	0	0	0	0	0	24	0	0	1	0	0	0	0
	GEND	0	0	1	0	0	0	0	4	0	1	0	0	0	0
	ISLA	0	0	6	2	45	0	2	0	221	25	3	5	10	9
	NA	0	0	10	0	34	0	5	0	19	115	3	2	5	12
	NAT	0	0	1	0	0	0	0	0	0	0	36	0	0	0
	PROT	0	0	0	0	3	0	0	0	0	0	0	45	0	0
	SCAN	0	0	1	0	0	0	0	0	0	3	0	0	51	0
	SUSP	0	0	0	0	0	0	0	0	0	0	0	0	0	45

Table A3: Distribution of categories in the consensus data.

**Category**	**Frequency**
APOC	24
ASEM	8
CONS	61
DIST	11
ELIT	397
ESO	1
EXPO	25
GEND	6
ISLA	328
NA	205
NAT	37
PROT	48
SCAN	55
SUSP	45
total	1,251

Confusion matrices and model statistics generated with R package *caret* (v6.0-92), additional statistics computed with ReCal (v. 0.1 Alpha).

Overall statistics for Model 1 - *Agreement on coded sentences between two coders*: N Agreements: 1251, N Disagreements: 1243, N Cases: 2494, Accuracy: 0.502, Krippendorff's Alpha (nominal): 0.416.

Overall statistics for Model 2 - *Agreement between human consensus and dictionary as coder*: N Agreements: 928, N Disagreements: 323, N Cases: 1251, Accuracy: 0.742, 95% CI: (0.717, 0.766), No Information Rate: 0.3173, Krippendorff's Alpha (nominal): 0.682.

Figure A1: RPC-Lex categories in the Facebook corpus over time (alternative plot).

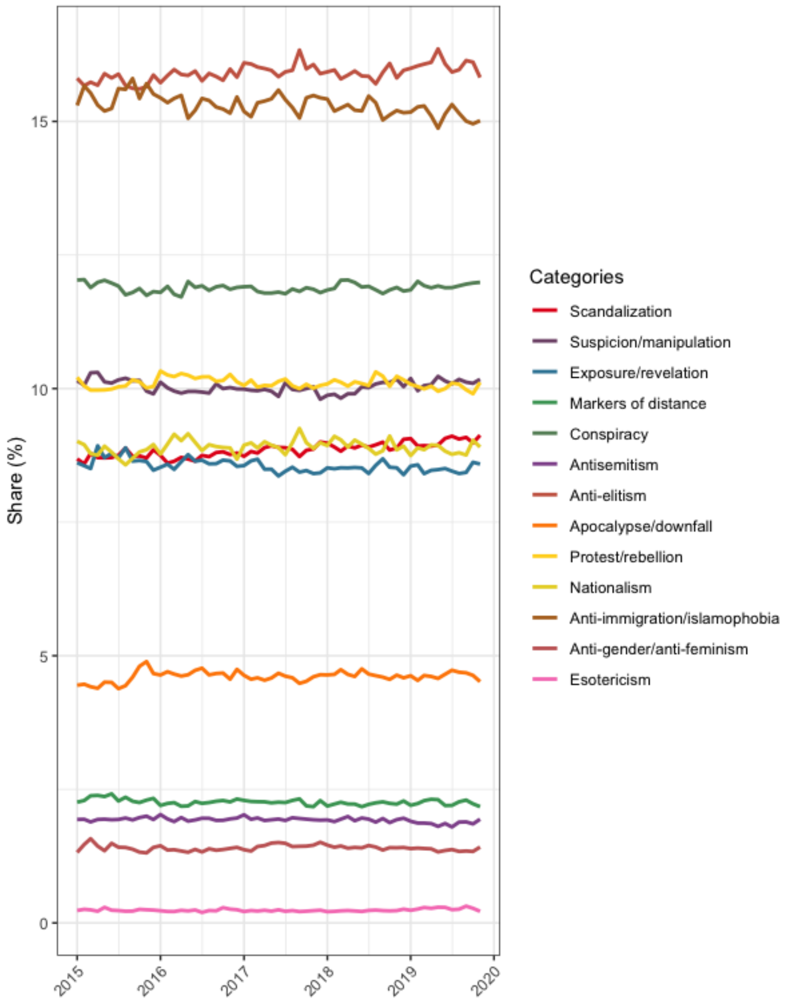

Supplement: Supplemental Material - RPC-Lex: A dictionary to measure German right-wing populist conspiracy discourse online [file sj-pdf-1-con-10.1177_13548565221109440.pdf]
